# The engineering principles of combining a transcriptional incoherent feedforward loop with negative feedback

**DOI:** 10.1186/s13036-019-0190-3

**Published:** 2019-07-10

**Authors:** Gregory T. Reeves

**Affiliations:** Department of Chemical and Biomolecular Engineering, North Carolina State University, Raleigh, NC 27695 USA

**Keywords:** Incoherent feedforward loop, Negative feedback, Gene regulation, Network motif, Engineering principles

## Abstract

**Background:**

Regulation of gene expression is of paramount importance in all living systems. In the past two decades, it has been discovered that certain motifs, such as the feedforward motif, are overrepresented in gene regulatory circuits. Feedforward loops are also ubiquitous in process control engineering, and are nearly always structured so that one branch has the opposite effect of the other, which is a structure known as an “incoherent” feedforward loop in biology. In engineered systems, feedforward control loops are subject to several engineering constraints, including that (1) they are finely-tuned so that the system returns to the original steady state after a disturbance occurs (perfect adaptation), (2) they are typically only implemented in the combination with negative feedback, and (3) they can greatly improve the stability and dynamical characteristics of the conjoined negative feedback loop. On the other hand, in biology, incoherent feedforward loops can serve many purposes, one of which may be perfect adaptation. It is an open question as to whether those that achieve perfect adaptation are subject to the above engineering principles.

**Results:**

We analyzed an incoherent feedforward gene regulatory motif from the standpoint of the above engineering principles. In particular, we showed that an incoherent feedforward loop Type 1 (I1-FFL), from within a gene regulatory circuit, can be finely-tuned for perfect adaptation after a stimulus, and that the robustness of this behavior is increased by the presence of moderate negative feedback. In addition, we analyzed the advantages of adding a feedforward loop to a system that already operated under negative feedback, and found that the dynamical properties of the combined feedforward/feedback system were superior.

**Conclusions:**

Our analysis shows that many of the engineering principles used in engineering design of feedforward control are also applicable to feedforward loops in biological systems. We speculate that principles found in other domains of engineering may also be applicable to analogous structures in biology.

**Electronic supplementary material:**

The online version of this article (10.1186/s13036-019-0190-3) contains supplementary material, which is available to authorized users.

## Background

**Fig. 1:**
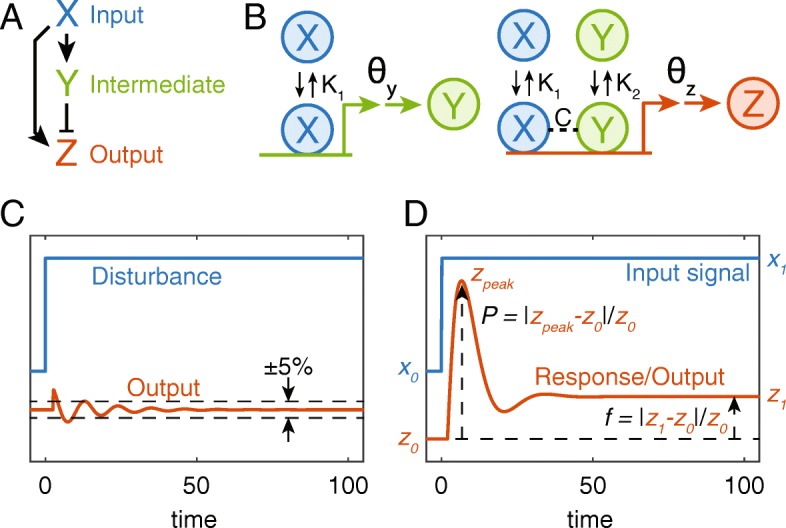
Incoherent feed-forward loops in biology and engineering. **a** In biology, a Type 1 incoherent feedforward loop (I1-FFL) is characterized as an input, X, which activates both an intermediate (Y) and the ultimate output (Z), while Y represses or inhibits Z. **b** Illustration an I1-FFL gene regulatory motif. Input X binds to the regulatory regions of both Y and Z with affinity *K*_1_, and Y binds to the regulatory region of Z with affinity *K*_2_. X and Y may bind the regulatory region of Z cooperatively with a multiplicative factor *C*. **c** Illustration of feedforward control in chemical engineering. The goal of process control is to minimize the response to a disturbance. Well-tuned feedforward control will result in only minimal change to the output upon a disturbance. **d** Illustration of the dynamics of an I1-FFL (modeled as described in B) in biology. Here, the goal is not to be completely insensitive to the input, X, but is often to have a sharp response (*P* large), followed by adaptation (*f* small)

Biological processes at the cell and tissue level are often controlled by complex networks of many interacting parts, such as neuronal networks, enzymatic networks, and gene regulatory networks, which themselves are composed of a number of overrepresented sets of interactions called “motifs” [1–3]. In gene regulation, the feedforward loop (FFL) – which consists of an input gene (X) that regulates an intermediate gene (Y), while both X and Y regulate an output gene (Z) – is one such overrepresented motif [1, 3–6]. One flavor of FFL, called an incoherent FFL (IFFL), occurs when the direct regulation of Z by X is in opposition to indirect regulation of Z by X through Y (see, for example, Fig. 1a). The IFFL has been widely studied, and it has been discovered to have a diverse array of roles, such as a mechanism to generate pulses, accelerate responses, detect fold changes, buffer noise, or achieve perfect adaptation [7–17]. While in this paper, we focus on IFFLs that can generate near-perfectly adapting pulses, we also briefly discuss some of the other relevant phenotypes listed above.

In biological IFFLs that act as a pulse generators, the concentration of Z has a strong, transient response to a change in the input, which is then dampened to a new steady state through the delayed action of Y. This strong, transient peak can be seen as a performance objective; that is, the input is a signal that the cell is designed to respond to, albeit transiently. If the new steady state concentration of Z is the same as before the change in input, the IFFL has achieved perfect adaptation (PA) [12, 13, 16, 18]. Previous studies have noted the IFFL motif can achieve PA in a wide range of model parameter values [12, 16, 19]. However, these models rely on extreme values of the model parameters – to ensure saturation of some responses and linear behavior of others – which may result in an array of biologically unacceptable phenotypes.

In contrast, in process control engineering, X is considered a “disturbance” that upsets the system, and the main goal of process control is to ensure Z is maintained at set point in the face of typical disturbances [20]. In particular, feedforward control loops, which use the IFFL structure, are designed to completely reject the effects of X on Z, during both the steady state and the transient. However, feedforward (FF) control alone can easily deviate from its objectives if model parameters are inaccurate. Combining it with feedback (FB) control (FF/FB circuit) can alleviate this problem. On the other hand, FB control alone is beset with dynamical instabilities and difficulty in achieving PA; a combined FF/FB system can achieve PA while improving stability.

Given these principles derived from process control in engineering disciplines, we asked whether the same engineering principles apply in biological systems [20–22]. First, we analyzed a previously reported IFFL gene network motif (Fig. 1a, b) [12]. We showed that near-perfect adaptation can be achieved under finely-tuned conditions and is highly sensitive to exact parameter values. As described above, the next step would be to investigate whether the addition of negative feedback may increase the range of acceptable parameter values of near-perfect adaptation. However, negative feedback loops have not been widely reported in transcriptional networks [4, 5]. Therefore, we analyzed the available data of transcriptional regulatory interactions in *E. coli* and identified a sizable number of transcriptional negative feedback loops, including one embedded within an I1-FFL. Once we established the prevalence of transcriptional negative feedback, we used the model to show that the addition of negative feedback improves the robustness of the near-perfect adaptation response. Finally, we showed that the combined feedforward/feedback (FF/FB) structure is also superior in terms of stability and achieves adequate compromise on peak response. We speculate that future studies aimed at discerning whether engineering principles of human-designed systems are found in analogous biological systems will be highly valuable.

## Methods

### Derivation and scaling of the model of transcriptional regulation

The model equations are modified from [12], in which the input, X, activates both Y and Z, while Y represses Z. As a convention throughout this work, the capital letter denotes the identity of the species, and the lowercase letter denotes the concentration of the species.

$$ \frac{d\hat{y}}{dt}={\beta}_y{f}_y\left(\frac{\hat{x}\left(t-{\theta}_y\right)}{{\hat{K}}_1}\right)-{\alpha}_y\hat{y} $$

$$ \frac{d\hat{z}}{dt}={\beta}_z{f}_z\left(\frac{\hat{x}\left(t-{\theta}_z\right)}{{\hat{K}}_1},\frac{\hat{y}\left(t-{\theta}_z\right)}{{\hat{K}}_2}\right)-{\alpha}_z\hat{z} $$

Where $\hat{s}$ is the variable that denotes the concentration of species S, *β*_*s*_ and *α*_*s*_ are parameters that dictate the rates of production and first-order degradation of species S, and:

1 $$ {f}_y(a)=\frac{a}{1+a},{f}_z\left(a,b\right)=\frac{a}{1+a+b+ ab/C}, $$

To rescale the equations, we let $x\equiv \hat{x}/{x}_0$, $y\equiv \hat{y}/\overline{y}$, and z̄ $\equiv \hat{z}/\overline{z}$, where *x*_0_ is the initial value of $\hat{x}$, $\overline{y}={\beta}_y/{\alpha}_y$, and $\overline{z}={\beta}_z/{\alpha}_z$. This results in the following scaled equations:

2 $$ {\tau}_y\frac{dy}{dt}={f}_y\left(\frac{x\left(t-{\theta}_y\right)}{K_1}\right)-y, $$

3 $$ {\tau}_z\frac{dz}{dt}={f}_z\left(\frac{x\left(t-{\theta}_z\right)}{K_1},\frac{y\left(t-{\theta}_z\right)}{K_2}\right)-z, $$

Where $$ {K}_1={\hat{K}}_1/{x}_0 $$, $$ {K}_2={\hat{K}}_2/\overline{y} $$, *τ*_*y*_ = 1/*α*_*y*_, and *τ*_*z*_ = 1/*α*_*z*_.

In all analysis in this paper, we set *x*(*t* < 0) ≡ *x*_0_ = 1, and we assume that the other two variables are at steady state: *y*(*t* < 0) = *y*_0_, *z*(*t* < 0) = *z*_0_. We assume they are at steady state regardless of the stability of that steady state. At time *t* = 0, *x* experiences a shift from *x* = *x*_0_ = 1 to *x* = *x*_1_ (usually equal to 10), which induces a change in both *y* and *z* (see Fig. 1). The steady states of *y*, *z* for *x* = *x*_1_ are defined as *y*_1_, *z*_1_, respectively.

### Design rule for perfect adaptation (PA)

To derive the design rule for PA, we analyze the system under the constraint that *z*_1_ = *z*_0_. At *x* = *x*_0_:

$$ {y}_0=\frac{x_0/{K}_1}{1+{x}_0/{K}_1},{z}_0=\frac{x_0/{K}_1}{1+{x}_0/{K}_1+{y}_0/{K}_2+{x}_0{y}_0/{K}_{12}} $$

Now at *x* = *x*_1_:

$$ {y}_1=\frac{x_1/{K}_1}{1+{x}_1/{K}_1},{z}_1=\frac{x_1/{K}_1}{1+{x}_1/{K}_1+{y}_1/{K}_2+{x}_1{y}_1/{K}_{12}} $$

Equating 1/*z*_1_ to 1/*z*_0_:

$$ \frac{1+{x}_1/{K}_1+{y}_1/{K}_2+{x}_1{y}_1/{K}_{12}}{x_1/{K}_1}=\frac{1+{x}_0/{K}_1+{y}_0/{K}_2+{x}_0{y}_0/{K}_{12}}{x_0/{K}_1} $$

Isolating the terms with *K*_12_ onto the LHS:

4 $$ \frac{K_1}{K_{12}}\left({y}_1-{y}_0\right)=\left(1+\frac{K_1}{x_0}+\frac{K_1{y}_0}{K_2{x}_0}\right)-\left(1+\frac{K_1}{x_1}+\frac{K_1{y}_1}{K_2{x}_1}\right), $$

OR:

5 $$ {K}_{12}^{PA}=\frac{y_1-{y}_0}{\frac{1}{x_0}\left(1+\frac{y_0}{K_2}\right)-\frac{1}{x_1}\left(1+\frac{y_1}{K_2}\right)}, $$

*C*_*PA*_ is defined as ${K}_{12}^{PA}/\left({K}_1{K}_2\right)$.

### Negative feedback

To add negative feedback, let there be W such that Z activates W, but W represses Z. The equation for the concentration of W, $\hat{w}$, is:

$$ \frac{d\hat{w}}{dt}={\beta}_w{f}_w\left(\frac{\hat{z}\left(t-{\theta}_w\right)}{{\hat{K}}_4}\right)-{\alpha}_w\hat{w} $$

Where

6 $$ {f}_w(a)=\frac{a}{1+a}, $$

Rescaling W in a similar manner to Y and Z, we arrive at:

7 $$ {\tau}_w\frac{dw}{dt}={f}_w\left(\frac{z\left(t-{\theta}_w\right)}{K_4}\right)-w, $$

With the addition of W, the form of the equation for *z* stays the same, but with an updated expression for *f*_*z*_:

8 $$ {f}_z\left(\frac{x}{K_1},\frac{y}{K_2},\frac{w}{K_3}\right)=\frac{\frac{x}{K_1}}{1+\frac{x}{K_1}+\frac{y}{K_2}+\frac{xy}{\left(C{K}_1{K}_2\right)}+\frac{w}{K_3}+\frac{xw}{\left({K}_1{K}_3\right)}+\frac{yw}{\left({K}_2{K}_3\right)}+\frac{xy w}{\left(C{K}_1{K}_2{K}_3\right)}}, $$

For simplicity, we have assumed the only cooperativity is between X and Y. Cooperativity between other components was analyzed in Additional file 1. It can be shown that the PA constraint for the FF/FB system reduces to the same constraint on *K*_12_, given *K*_1_, *K*_2_, *x*_1_.

### Analysis of the RegulonDB data set

Two flat files from the RegulonDB database that contain (1) the names of TF complexes and the genes they regulate and (2) the names of TF complexes and the genes that encode the constituents of those complexes were used to create a matrix of interactions among the TF complexes. The rows denoted the regulators, and the columns the regulatees. Each element of the matrix was either a zero (for no regulation), a “+ 1” (for positive regulation), a “-1” (for negative regulation), a “2” (for mixed, or dual regulation), or a “3” (for regulation of unknown sign). The matrix was searched for pairs of off-diagonal elements such that both element *M*(*i*, *j*) ≠ 0 and element *M*(*j*, *i*) ≠ 0 (see Additional file 2). Seventeen such pairs were found (see Additional file 2). Of these, nine were definitively negative feedback (one element was − 1, the other was + 1) and three were mixed, in which at least one element was a 2. Of these 12, one pair was such that the positive regulator was also the end node of two I1-FFLs (see Additional file 1 and Additional file 2).

## Results and discussion

### A model of incoherent feedforward transcriptional regulation

There are four varieties of incoherent feedforward (FF) control in a three-node system [5, 23]. In this paper, we study the incoherent feedforward loop Type 1 (I1-FFL) motif, in which the input signal (X) activates both the intermediate (Y) and the ultimate output (Z), while Y represses Z (Fig. 1a) [12]. Our model is one of transcriptional regulation, so that X represents an input transcription factor, which can bind to the *cis*-regulatory regions of Y and Z, with affinity *K*_1_ (Fig. 1b). This results in the transcription and translation of Y (double arrows in Fig. 1b), which is also a transcription factor that binds to the regulatory region of Z with affinity *K*_2_. In our model, the binding of X and Y is cooperative (dashed line in Fig. 1b), so that their synergistic binding is described by *K*_12_ = *CK*_1_*K*_2_. Cooperative binding may also take place between other components; however, cooperativity other than that between X and Y is not required (see Additional file 1 for analysis of cooperativity). Therefore, we retain only the one necessary cooperative term to test our hypotheses, and thus our main analysis assumes all other binding interactions are non-cooperative.

In process control engineering, X is seen as a disturbance to the system; the goal of standard FF control (which uses an IFFL motif) is to reject the effect that X has on Z. If the FF controller is tuned properly, the output variable is maintained at or near its desired value (e.g., within 5%), both in the transient and in the ultimate output (Fig. 1c). In contrast, in biological systems, X is seen as an input signal, and the I1-FFL motif is often designed so that the levels of Z exhibit a transient response, then return (close to) their original value (Fig. 1d). Thus, the peak height and the difference between initial and final levels of Z represent performance metrics (Fig. 1d).

Here we model the I1-FFL as a system of delay differential equations, which are an extension of the model from [12] (Eqs. 1–3 in Methods). Note that X activates both Y and Z, with binding affinity *K*_1_, while Y represses Z (even if X is also bound) with binding affinity *K*_2_. The final term in the denominator of *f*_*z*_ is the cooperativity term (*xy*/*K*_12_ = *xy*/(*CK*_1_*K*_2_)). See Methods for more details on model derivation.

In this paper, we hold *x* = *x*_0_ = 1 for *t* < 0, which results in an initial steady state of *y*_0_ and *z*_0_. At time *t* = 0, *x* experiences a step increase: *x*(*t* ≥ 0) = *x*_1_ > 1, which results in an initial increase in both *y* and *z*. However, after some initial transient, the increase in *y* also begins to repress *z*. For most values of the parameters, this results in a peak value of *z* (*z*_*peak*_), followed by an adaptation back to a new steady state value, *z*_1_ (Fig. 1d). Here, we have normalized the peak level and the adaptation metric as *P* ≡ (*z*_*peak*_ − *z*_0_)/*z*_0_ and *f* = (*z*_1_ − *z*_0_)/*z*_0_, respectively (see Fig. 1d). For the rest of the paper, we will take *τ*_*y*_ = *τ*_*z*_ = 1, and *θ*_*y*_ = *θ*_*z*_ = 0.5.

### The type I incoherent feedforward loop can be tuned for perfect adaptation

Previous analysis of the above model (Eqs. 1–3) showed that I1-FFL transcriptional regulation can, under the right conditions, act as a fold-change detector (FCD) [12]. One of the necessary conditions for a model to act as a FCD is near-perfect adaptation (NPA), which occurs when the final state, *z*_1_, is within a few percent of the initial state, *z*_0_ (e.g., |*f*| ≤ *ε* = 0.05). Whereas previous work analyzed the model (Eqs. 1–3) from the standpoint of FCD, the model (and I1-FFLs generally) can produce several other phenotypes, including the less restrictive phenotypes of adaptation and pulse generation.

**Fig. 2:**
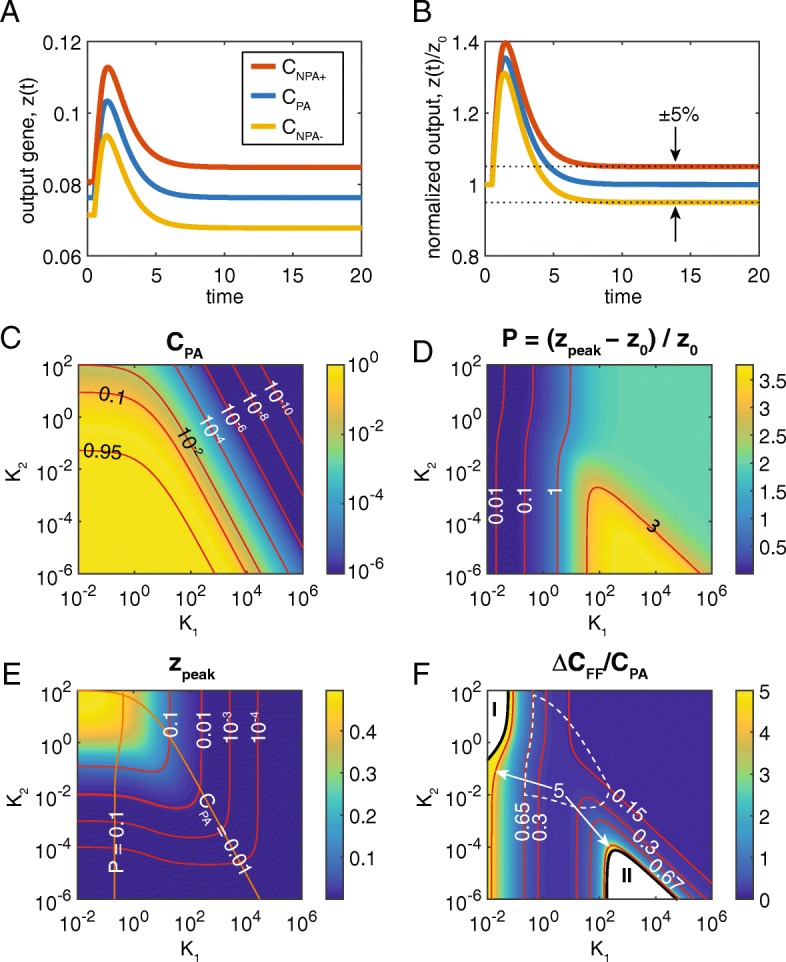
Tuning the I1-FFL for near perfect adaptation. **a** Dynamics of the output, *z*(*t*), upon a step-increase in *x* from 1 to 10 at time *t* = 0. Blue curve: perfect adaptation. Red and yellow curves: near-perfect adaptation, such that |*f*| = 0.05. **b** Normalized output of *z*(*t*). When the curves are normalized, it is clear that the red and yellow curves achieve near-perfect adaptation. **c** Heatmap of the value of *C* required for perfect adaptation given *K*_1_, *K*_2_, for a step increase in *x* from 1 to 10. Red curves (here and elsewhere) represent contours whose values are indicated directly on plot. Note that axis limits for *K*_1_ range from strong (10^− 2^) to very weak (10^6^) affinity, while those for *K*_2_ range from very strong (10^− 6^) to weak (10^2^). **d** Heatmap of peak metric, *P*. As *K*_1_ becomes too low, this metric drops to unacceptably low values. **e** Absolute levels of the peak in output. Orange curves: *P* = 0.1 and *C*_*PA*_ = 0.01. These curves, together with the *z*_*peak*_ = 0.01 contour, delimit a biologically realistic region of the parameter space. **f** Heatmap of the normalized range of *C* values under which NPA is achieved. Black curves delimit two regions in which NPA is achievable with any value of *C*. In Region I, *Y* and *Z* are decoupled, and change only slightly upon increase in *X*. Region II is described in [12] as a FCD region, which requires NPA. In the biologically realistic region of the parameter space, the values of *C* under which NPA can be achieved ranges from 13 to 65% of *C*_*PA*_

Our model analysis shows that it is possible to tune the FFL such that perfect adaptation (PA) is achieved. In other words, *z*_1_ = *z*_0_ (blue curve in Fig. 2a, b). This condition obtains when, for given *K*_1_, *K*_2_, the value of *C* is equal to *C*_*PA*_, which is given by Eq. 5 (see Methods). If the value of *C* deviates slightly from *C*_*PA*_, PA is lost, but NPA may be maintained. Here we define *C*_*NPA*+_ and *C*_*NPA*−_ as the values of *C* in which *f* =  + *ε* and –*ε*, respectively (red and gold curves in Fig. 2a, b). For most of this work, we have set *ε* = 0.05; however, our results are not materially affected by the precise value of *ε* (see Additional file 1).

For *x*_1_ = 10, a heatmap of *C*_*PA*_ values, as a function of *K*_1_, *K*_2_, is depicted in Fig. 2c. Note that cooperativity is required to achieve PA: *C*_*PA*_ < 1, although it approaches 1 for *K*_1_, *K*_2_ ≪ 1 (see Additional file 1 and Fig. 2c). As *C* represents a fold-change cooperativity parameter, values of *C* < 1 represent positive synergy: when X is bound to the regulatory region of Z, it enhances the ability of Y to bind, and vice versa. A lower bound for reported values of *C* in other systems is on the order of 0.01 [24–28]. Therefore, as models of biological processes must be constrained to biologically realistic phenotypes, it is unlikely that PA can be achieved in the upper right region of parameter space (Fig. 2c).

### Moderate values of parameter space correspond to biologically realistic phenotypes

Given the rough lower bound on *C*, it is reasonable to ask what other constraints can be put on the model. We impose two additional biologically realistic constraints on the model. First, the relative peak size, *P* = (*z*_*peak*_ − *z*_0_)/*z*_0_, should be greater than 0.1 to ensure a quality signaling response. A heatmap of *P* as a function of *K*_1_, *K*_2_ is depicted in Fig. 2d. The *P* = 0.1 contour resides at roughly *K*_1_ = 0.25; this implies that, if X binds the regulatory region of Z too tightly, a 10-fold increase in *x* (from 1 to 10) does not produce a strong peak, as the Z promoter is already saturated, even at low values of *x*.

Second, the absolute peak in *z* must be greater than 0.01 (Fig. 2e). As our model is scaled such that the maximum possible value of *z* is 1, this corresponds to 1% of the maximum possible concentration of *z*, given the promoter strength and degradation rate. We take *z*_*peak*_ = 0.01 to be the minimum value in order to achieve a biologically detectable signal; however, one may relax this minimum value somewhat, which would not significantly affect our results. Taking these three constraints on *C*_*PA*_, *P*, and *z*_*peak*_, a small region of *K*_1_, *K*_2_ parameter space is deemed “biologically realistic.” For context, all three contours are plotted in Fig. 2e. Again, this region could be modified if other values of the “biologically realistic” constraints are chosen. It should also be noted that the biologically realistic region also delimits the region of parameter space in which the I1-FFL can act as a pulse generator or perfect adaptor (see Additional file 1).

### Sensitivity of NPA with respect to I1-FFL parameters

Robustness is an additional objective that is often imposed on biological systems [29–34]. Therefore, we analyzed the range of values *C* about *C*_*PA*_ for which NPA is maintained. We define Δ*C*_*FF*_ ≡ *C*_*NPA*+_ − *C*_*NPA*−_, and plotted a heatmap of Δ*C*_*FF*_/*C*_*PA*_ in Fig. 2f (see Additional file 1 for derivation of *C*_*NPA*+_ and *C*_*NPA*−_). There are two regions of the *K*_1_, *K*_2_ parameter space in which NPA is achieved regardless of the value of *C* (see Additional file 1 and Fig. 2f), neither of which reside in the biologically realistic region of parameter space (white dashed region in Fig. 2f). In Region I, the value of *K*_1_ is sufficiently low such that, even at *x*_0_ = 1, the regulatory regions of both Y and Z are saturated with X, and thus, Y and Z are relatively insensitive to X (see Additional file 1). Furthermore, in Region I, *K*_2_ is large enough that Y has little influence on Z. Thus, an increase in *x* does not appreciably change the value of *z*, which means that while *z*_1_ ≈ *z*_0_ (so that |*f*| < *ε*), there is no peak in the value of *z* (see Additional file 1: Figure S1). This scenario cannot truly be described as NPA. Indeed, the nodes of the IFFL are decoupled in this region, so that the biological phenotype, or function, of the IFFL is indistinct from two nodes acting independently of one another.

Region II has previously been reported as not only the region in which NPA is easily achieved, but also where the I1-FFL can act as a fold-change detector (Additional file 1: Figure S2A) [12]. In this regime, in which *K*_1_ ≫ 1, *K*_2_ ≪ 1, and *K*_1_*K*_2_ < 1, binding of X is in the linear regime, and *z* depends on the ratio of *x*/*y* [12]. On the other hand, the absolute response of Z is limited to 0.01% of its maximum (Additional file 1: Figure S2B).

In contrast, in the biologically realistic region of parameter space, Δ*C*_*FF*_ is limited to 13% -- 65% of *C*_*PA*_ (Fig. 2f). For example, for *K*_1_ = 1, *K*_2_ = 0.1, to maintain the objective of NPA, the system can tolerate only a ∼ 30% perturbation to *C*_*PA*_. In other words, the I1-FFL model is relatively sensitive to changes to *C* where NPA is concerned. To increase the robustness of the system, we considered a combined feedforward/feedback (FF/FB) system.

### Transcriptional negative feedback cycles

In engineering, the sensitivity of the feedforward controller algorithm with respect to model parameters can be mitigated by the action of a negative feedback loop (see Eqs. 6–8 in Methods). Indeed, negative feedback has been shown to confer robustness of I1-FFL performance objectives, such as rise time and decay time, in a simplified model of gene regulation [35]. However, in contrast to the high frequency of transcriptional FFL motifs, it has been previously reported that no transcriptional cycles (beyond autoregulation) exist in the model organism *E. coli* [4]. Further studies suggest that transcriptional negative feedback is rare, possibly due to the sluggish nature of double-transcriptional loops, while acknowledging the widespread use of mixed negative feedback loops in which one branch is transcriptional and the other branch is based on protein-protein interactions [5, 36]. To investigate further, we analyzed a recently-updated data set of *E. coli* transcriptional interactions, and found 17 instances of cycles of length 2, five of which are positive feedback, nine of which are negative feedback, and three of which are mixed [37]. Of these interactions, one of the negative feedback cycles was embedded within multiple I1-FFL structures (see Additional file 2).

### A combined feedforward/feedback system is more robust than one with feedforward alone

**Fig. 3:**
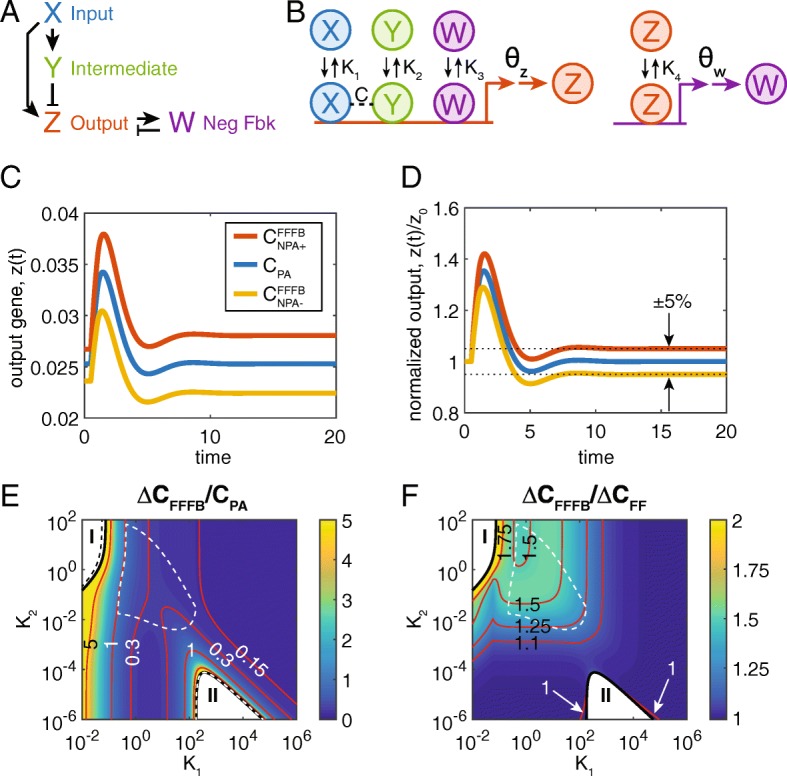
Behavior of the combined FF/FB system. **a** The addition of W to the network motif results in a negative feedback loop involving Z and W. **b** Illustration of the mechanism of negative feedback. Z activates W and is repressed by W. Illustration of Y not shown (see Fig. 1b). **c** Dynamics of the output, *z*(*t*), upon a step-increase in *x* from 1 to 10 at time *t* = 0 for the combined FF/FB system. Blue curve: perfect adaptation. Red and yellow curves: near-perfect adaptation, such that |*f*| = 0.05. **d** Normalized output of *z*(*t*). When the curves are normalized, it is clear that the red and yellow curves are tuned for near-perfect adaptation. The value of *C* needed to achieve NPA is more distant from *C*_*PA*_ than for the FF only system. **e** Heatmap of the normalized range of *C* values under which NPA is achieved. Black curves delimiting Regions I and II are analogous to those described in Fig. 2. In the biologically realistic region (defined for the FF/FB system), the range of *C* values under which NPA can be achieved ranges from 25 to 100% of *C*_*PA*_. **f** Heatmap of the ratio of ranges of *C* values for which NPA is achieved for the FF/FB system vs the FF only system. The addition of a FB loop increases the range of values of *C* by 21--54%

Given the prediction regarding the efficacy of a FB loop to extend the robustness of NPA in an IFFL motif, we investigated a combined FF/FB system. We added a fourth node, W, which is activated by Z, and represses Z (Fig. 3a, b). These interactions introduce multiple new parameters to the model (see Methods), including two affinity binding parameters (*K*_3_, *K*_4_) and a transcription/translation delay in W (*θ*_*w*_; see Fig. 3b).

The transient of the FF/FB system (Fig. 3c, d) behaves similarly to the FF only system (cf. Figure 2a, b). A strong peak is initially experienced on a step change in *x* from 1 to 10, and, with the proper tuning of the FF loop, *z* returns to its initial value. Furthermore, the value of *C* required for PA is the same as in the FF only system, and depends only on *K*_1_, *K*_2_ (and not *K*_3_, *K*_4_; see Additional file 1). However, the presence of the negative FB loop alters the values of *C* that give NPA (denoted ${C}_{NPA+}^{FFFB}$ and ${C}_{NPA-}^{FFFB}$ see Additional file 1 and Fig. 3c, d).

We calculated the value of $\Delta {C}_{FFFB}\equiv {C}_{NPA+}^{FFFB}-{C}_{NPA-}^{FFFB}$ for *K*_3_, *K*_4_ = 0.1 (moderate negative feedback) and varying values of *K*_1_, *K*_2_ (see Fig. 3e). Compared to the FF only system, the combined FF/FB system has a wider range of *C* values that admit NPA (compare Fig. 3e to 2d). The meanings of Regions I and II remain the same, although Region I is a bit larger in the FF/FB system (compared to dashed black curve in Fig. 3e, which represents the FF only Region I), while Region II remains effectively the same size (see Additional file 1 for further discussion of Regions I and II).

To directly compare the two systems, we plotted the ratio Δ*C*_*FFFB*_/Δ*C*_*FF*_ in Fig. 3f. For the range of biologically realistic values, Δ*C*_*FFFB*_ is 21% -- 54% larger than Δ*C*_*FF*_ (ratios of 1.21 -- 1.54). (Note that the biologically realistic region shown in Figs. 3e, f is for the FF/FB system; see Additional file 1: Figure S3.) Indeed, with the exception of a small region of *K*_1_, *K*_2_ parameter space (near Region II), the FF/FB system is always superior to the FF only system (ratio greater than one). Given the advantage of the FF/FB system with respect to NPA objectives, we next investigated whether the dynamic properties of the FF/FB system were also advantageous.

As mentioned previously, in our analysis, we attempted to retain only the interactions that were necessary to explain the NPA phenotype its robustness. As such, only the interactions between X and Y included cooperativity. We may also incorporate cooperativity between X and W, or Y and W, which does not qualitatively affect our result that adding the FB module to the I1-FFL increases the robustness of the system (Additional file 1: Figure S4). Additionally, our results do not depend on the exact choice of *ε* (Additional file 1: Figure S5).

### Dynamic analysis reveals the FF/FB system is superior to the one with FB alone

**Fig. 4:**
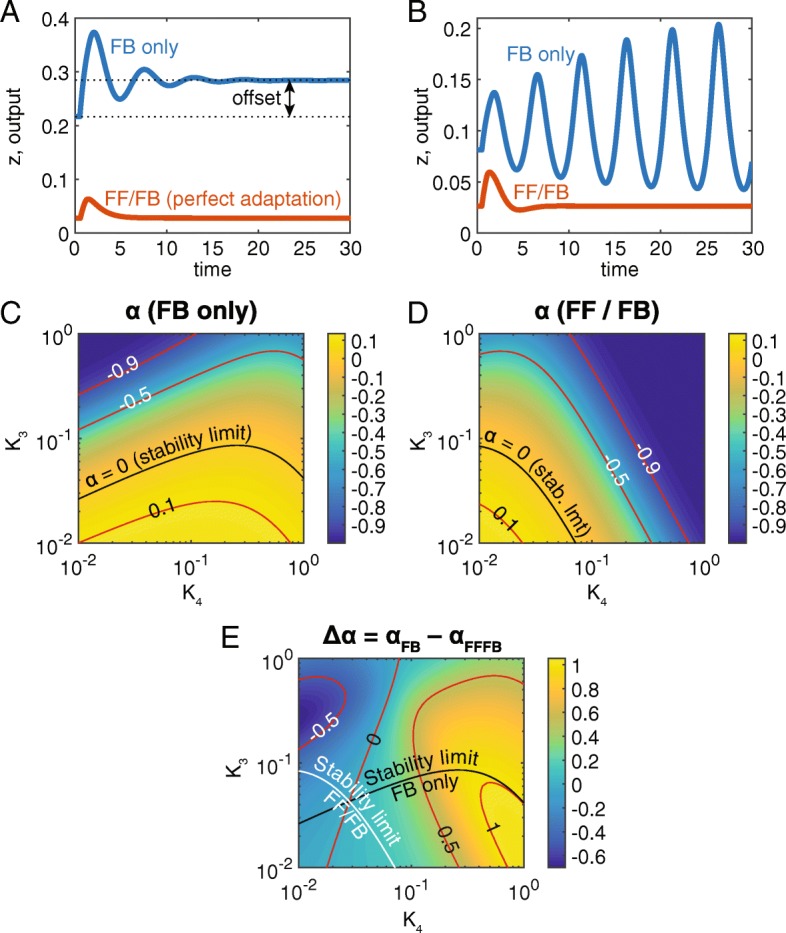
Dynamics of the FB only and combined FF/FB systems. **a** A (proportional) negative feedback alone (blue curve), upon perturbation, always results in offset from the original value. A comparable combined FF/FB system also may exhibit offset; however, the I1-FFL in the FF/FB system can be tuned for perfect adaptation. The FF/FB system may also have a more rapid approach to steady state. **b** The addition of an I1-FFL to the FB system may result in more stable dynamics. For this choice of parameters, the FB only system is unstable, while the FF/FB system is stable. **c** Heatmap of the real part of principal eigenvalue (α) for the FB only system. Black curve is the contour for which *α* = 0, which indicates the stability limit of the FB only system. Smaller values of *K*_4_ result in an unstable system (see part B). Here and elsewhere, values of *α* for other contours (red curves) are indicated directly on plot. **d** Same as C, but for the combined FF/FB system. **e** Heatmap of Δ*α*, the difference between the real parts of the principal eigenvalues for the two systems. On the right side of the zero contour, the values of Δ*α* are greater than zero, indicating the FF/FB system has a more rapid approach to steady state. Black and white curves indicate the stability boundaries for the FB only and FF/FB systems, respectively (see also parts C, D)

Negative feedback loops are beset by multiple drawbacks [38]. First, proportional feedback (which is the most common mechanism that naturally occurs biological systems) always results in offset (see Fig. 4a). In other words, after a disturbance upsets the system, the output does not return to its original value. In order to mitigate this, one may increase the strength of the negative feedback response. However, this often results in the second major drawback of negative feedback: dynamic instabilities and/or ringing oscillations can result from strong negative feedback, especially in systems with substantial delays (Fig. 4b) [38]. On the other hand, if the primary disturbance can be partially or fully rejected by a FFL, both drawbacks may be avoided (Fig. 4a, b). To investigate these principles in a gene regulation system, we compared the behavior of a FB only model with a combined FF/FB model. To illustrate dynamic instability, we set the Hill coefficient for all DNA binding interactions to *n* = 2 (see Additional file 1).

To determine the stability of both the FB only and combined FF/FB systems, we calculated *α*, the real part of the principal eigenvalue (see Additional file 1), for varying values of *K*_3_, *K*_4_ (for the FF/FB system, we set *K*_1_ = 1, *K*_2_ = 0.1; Fig. 4c, d). While neither system is clearly superior to the other, for moderate activation of W by Z (*K*_4_ ≈ 0.1 or greater), strong negative feedback (*K*_3_ < 0.1) tends to result in an unstable FB-only system, while the FF/FB system is always stable. Indeed, *α*_*FB*_ − *α*_*FFFB*_ > 0 for this region of moderate activation of W (Fig. 4e). Even when both systems are stable, *α*_*FFFB*_ < *α*_*FB*_ implies the FF/FB system reaches steady state faster (see, for example, Fig. 4a). However, adding a second layer of control can often result in trade-offs, where an advantage gained in one area results in a disadvantage in another. Therefore, we will compare the performance of the FB only and combined FF/FB models with regards to two other objectives: normalized peak, *P*, and absolute peak, *z*_*peak*_.

### A combined FF/FB system achieves compromise on multiple objectives

**Fig. 5:**
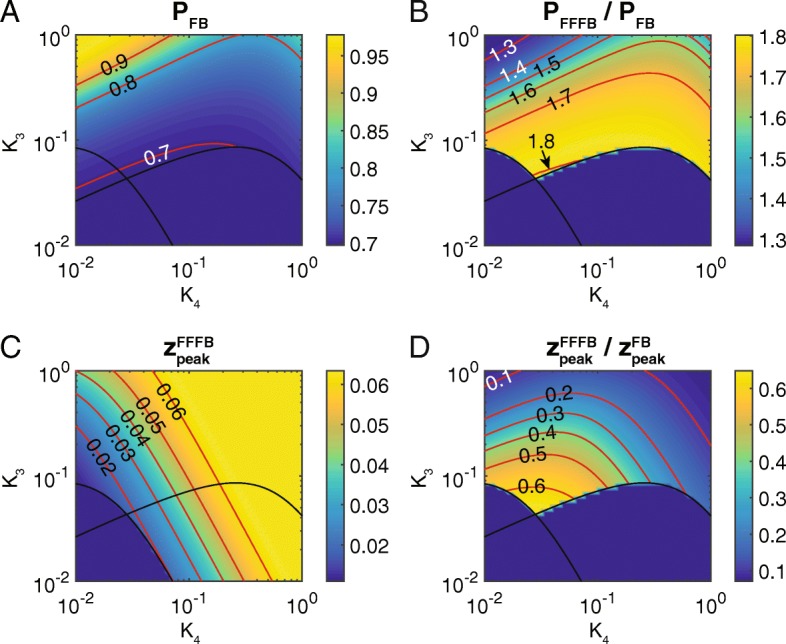
Comparison of performance metrics of the FB only system with the combined FF/FB system. **a** Heatmap of the normalized peak, *P*, for the FB only system. This parameter (and others defined in this figure) is undefined for unstable systems. **b** Ratio of *P* for the FF/FB system to that for FB only. According to this metric, the FF/FB system always outperforms the FB only system. However, the metric *P*_*FFFB*_ is roughly constant at 0.46, regardless of the values of *K*_3_, *K*_4_ (see Additional file 1). **c** Heatmap of the absolute peak, *Z*_*peak*_, for the FF/FB system and varying values of *K*_3_, *K*_4_. Note that the absolute peak value is, at best, 0.06. While this is low, it is adequate. **d** Ratio of the absolute peak for the FF/FB system to that for FB only. According to this metric, the FB only system always outperforms the FF/FB system (ratio less than one). However, given the other performance objectives for which the FF/FB system is superior, this is an acceptable trade-off

To compare the performance of the FF/FB system vs that of the FB only system, we calculated the peak value of *z* in both systems while varying *K*_3_, *K*_4_ from 0.01 to 1. First, we found the normalized peak, *P*, of the FB only system ranged from roughly 0.7 to 1 (Fig. 5a). On the other hand, the normalized peak for the FF/FB system is nearly independent of *K*_3_, *K*_4_ values and is roughly 1.256 (see Additional file 1: Figure S6). Therefore, the FF/FB system outperforms the FB only system on this metric as well: the FF/FB system is a 30–80% improvement over the FB only system (Fig. 5b).

One major drawback of the combined system is that, with two repressors of the output (Y and W), the levels of the output (with respect to its maximum possible levels) are low. For the parameter values chosen (*K*_1_ = 1, *K*_2_ = 0.1), *z*_*peak*_ remains above the lower bound of 0.01 (see Fig. 2e). However, this is significantly lower than the value of *z*_*peak*_ seen in the FB only system (Fig. 5d). In all, the combined FF/FB system is superior in its dynamics and normalized peak value, while compromising on the absolute peak value.

## Conclusions

Engineering principles form the bedrock of good design practices for human-built systems; the alternative is poorly functioning systems. In the same way, the fitness of biological systems is also somewhat dependent upon engineering principles similar to the ones we have discovered [20, 21]. And while biological systems are not designed in the way that human-built systems are, nevertheless, we can expect to find engineering principles in biological systems. As such, when analogies can be drawn to human systems, these analogies can serve as signposts for which engineering principles to expect [20]. *We have used this expectation as a guiding principle* in analysis of a I1-FFL system for gene regulation. Such network motifs are commonly found in biological systems, and have been analyzed extensively [4, 7, 8, 10–15, 35]. In particular, the I1-FFL system has been found to exhibit several design principles, including its ability to act as a response accelerator, fold-changedetector, or noise suppressor. In this paper, we focused on the phenotype of adaptation of a pulse generating I1-FFL.

In our analysis, we began with a previously discussed model of an I1-FFL motif [12]. We found that the near-perfect adaptation phenotype of the I1-FFL motif requires a finely-tuned level of cooperativity between the activator, X, and the intermediate node, Y. In a synthetically-designed system, this level of cooperativity may be difficult to alter, as it may be tied to the crystal structure of the transcription factors (see, for example, [39]). Another possibility would be to tune the affinity of the transcription factors for their DNA binding sites. This can be achieved by directed evolution or altering the binding site sequence. Even so, these approaches would simply alter values of the finely tuned parameters. We argue that a superior alternative, from our analysis of our model in light of engineering design, is to conjoin a negative feedback loop to the I1-FFL to increase the robustness of adaptation. Our results demonstrate that the combined FF/FB system has an increased range of possible parameter values that achieve near-perfect adaptation as compared to the FF-only system. In a similar manner, we analyzed a gene regulatory motif with proportional negative feedback, and found its offset and dynamics can both be improved by the presence of an I1-FFL, which is also a phenomenon seen in engineering.

Initial transcriptional network analysis in *E. coli* found no transcriptional negative feedback loops [4]. However, we now have experimental data for roughly twice as many transcription factors [37], and our resulting analysis revealed roughly a dozen negative feedback cycles of length 2. Therefore, transcriptional negative feedback is not yet a widely-studied phenomenon, and as such, our conclusions serve as a theory-driven prediction regarding expectations of I1-FFLs that may achieve perfect adaptation. Furthermore, it is possible that other objectives of the I1-FFL may also benefit from being combined with negative feedback, either transcriptional, as studied here, or through signaling factors or protein-protein interactions. We conclude that our understanding of gene regulatory motifs has benefitted from an engineering analysis. We also speculate that other areas of biology – in particular, those for which engineering principles of analogous human structures are known – may benefit from a similar analysis.

## Additional files

Additional file 1:  Supplemental procedures and supplemental figures. The supplemental procedures section includes Cooperativity is required for perfect adaptation, Cooperativity required for perfect adaptation becomes negligible if K_1,K_2≪1, Near-perfect adaptation for the FF only model, Regions I and II for the FF only model, Region I results from a simplification to the model for low K_1, moderate K_2, Near perfect adaptation for the combined FF/FB model, Regions I and II for the FF/FB model, Model equations with cooperativity between X and W, and/or between Y and W, Model equations and perfect adaptation for n=2, Derivation of the principal eigenvalue, and Analysis of RegulonDB data set. The supplemental figures include Fig. S1: Dynamic behavior of the model in Region I, Fig. S2: Analysis of the phenotypes in the K_1,K_2 plane, Fig. S3: Heatmap of z_peak for the FF/FB model, Fig. S4: Adding cooperativity to interactions between X and W, and/or between Y and W, Fig. S5: Varying ε, Fig. S6: Independence of P from K_3,K_4 for the FF/FB model. (DOCX 831 kb)

Additional file 2: Tables associated with RegulonDB analysis. The tabs of the excel file include M_total, M_TF, M_targets, FF_FB combined, and List of FB loops. (XLSX 2199 kb)

## Data Availability

Project name: Project home page: https://github.ncsu.edu/gtreeves/Reeves2019 Operating system(s): Platform independent. Programming language: Matlab.

## Abbreviations

FB: Feedback
FCD: Fold-change detection
FF: Feedforward
FF/FB: Combined feedforward/feedback
I1-FFL: Type 1 incoherent feedforward loop
IFFL: Incoherent feedforward loop
NPA: Near-perfect adaptation
PA: Perfect adaptation
