# Supplementary material for: The engineering principles of combining a transcriptional incoherent feedforward loop with negative feedback
Source: J Biol Eng. 2019 Jul 10;13:62. doi: 10.1186/s13036-019-0190-3 (PMC6617889; doi:10.1186/s13036-019-0190-3)
Supplement: Supplementary file 1 — Supplemental procedures and supplemental figures. The supplemental procedures section includes Cooperativity is required for perfect adaptation, Cooperativity required for perfect adaptation becomes negligible if K_1,K_2≪1, Near-perfect adaptation for the FF only model, Regions I and II for the FF only model, Region I results from a simplification to the model for low K_1, moderate K_2, Near perfect adaptation for the combined FF/FB model, Regions I and II for the FF/FB model, Model equations with cooperativity between X and W, and/or between Y and W, Model equations and perfect adaptation for n=2, Derivation of the principal eigenvalue, and Analysis of RegulonDB data set. The supplemental figures include Fig. S1: Dynamic behavior of the model in Region I, Fig. S2: Analysis of the phenotypes in the K_1,K_2 plane, Fig. S3: Heatmap of z_peak for the FF/FB model, Fig. S4: Adding cooperativity to interactions between X and W, and/or between Y and W, Fig. S5: Varying ε, Fig. S6: Independence of P from K_3,K_4 for the FF/FB model. (DOCX 831 kb) [file 13036_2019_190_MOESM1_ESM.docx]

Additional File 1: Supplemental material for “The engineering principles of combining a transcriptional incoherent feedforward loop with negative feedback”

Gregory T. Reeves

Department of Chemical and Biomolecular Engineering, North Carolina State University, Raleigh, NC 27695, [gtreeves@ncsu.edu](mailto:gtreeves@ncsu.edu)

# Supplemental inventory

## Supplemental methods

Cooperativity is required for perfect adaptation

Cooperativity required for perfect adaptation becomes negligible if $K_{1},K_{2}\ll1$

Near-perfect adaptation for the FF only model

Regions I and II for the FF only model

Region I results from a simplification to the model for low $K_{1}$, moderate $K_{2}$

Near perfect adaptation for the combined FF/FB model

Regions I and II for the FF/FB model

Model equations with cooperativity between X and W, and/or between Y and W

Model equations and perfect adaptation for $n=2$

Derivation of the principal eigenvalue

Analysis of RegulonDB data set

## Supplemental figures

Fig. S1: Dynamic behavior of the model in Region I

Fig. S2: Analysis of the phenotypes in the $K_{1},K_{2}$ plane

Fig. S3: Heatmap of $z_{peak}$ for the FF/FB model

Fig. S4: Adding cooperativity to interactions between X and W, and/or between Y and W

Fig. S5: Varying $\varepsilon$

Fig. S6: Independence of $P$ from $K_{3},K_{4}$ for the FF/FB model

# Supplemental methods

### Cooperativity is required for perfect adaptation

If we assume no cooperativity, then $C=1$ and $K_{12}=K_{1}K_{2}$. In that case, equating $1/{z_{1}}$ to $1/{z_{0}}$ gives us:

$$\frac{1+{x_{1}}/{K_{1}}+{y_{1}}/{K_{2}}+{x_{1}y_{1}}/\left( K_{1}K_{2} \right)}{{x_{1}}/{K_{1}}}=\frac{1+{x_{0}}/{K_{1}}+{y_{0}}/{K_{2}}+{x_{0}y_{0}}/\left( K_{1}K_{2} \right)}{{x_{0}}/{K_{1}}}$$

Isolating the terms with $K_{2}$ onto the LHS:

$$\frac{K_{1}y_{1}}{K_{2}x_{1}}-\frac{K_{1}y_{0}}{K_{2}x_{0}}+\frac{y_{1}-y_{0}}{K_{2}}=\left( 1+\frac{K_{1}}{x_{0}} \right)-\left( 1+\frac{K_{1}}{x_{1}} \right)$$

Simplifying the RHS and dividing both sides by $K_{1}$ and rearranging:

$$\frac{1}{K_{2}}\left( \frac{y_{1}}{x_{1}}-\frac{y_{0}}{x_{0}}+\frac{y_{1}}{K_{1}}-\frac{y_{0}}{K_{1}} \right)=\frac{1}{x_{0}}-\frac{1}{x_{1}}$$

OR:

$$\frac{1}{K_{2}}\left[ y_{1}\left( \frac{1}{x_{1}}+\frac{1}{K_{1}} \right)-y_{0}\left( \frac{1}{x_{0}}+\frac{1}{K_{1}} \right) \right]=\frac{1}{x_{0}}-\frac{1}{x_{1}}$$

However, the terms inside the rounded parenthesis can be simplified:

$$\frac{1}{x}+\frac{1}{K_{1}}=\frac{x+K_{1}}{xK_{1}}=\frac{1}{yK_{1}}$$

Thus, we have:

$$\frac{1}{K_{2}}\left[ \frac{y_{1}}{y_{1}K_{1}}-\frac{y_{0}}{y_{0}K_{1}} \right]=\frac{1}{x_{0}}-\frac{1}{x_{1}}$$

Clearly, the terms inside the square bracket cancel, which implies that if $C=1$, you cannot equate $1/{z_{1}}$ to $1/{z_{0}}$. In other words, regardless of the values chosen for $x_{1}$ and $K_{1}$, there is no value of $K_{2}$ that satisfies the PA constraint if $C=1$, and PA is impossible without cooperativity.

### Cooperativity required for perfect adaptation becomes negligible if $\boldsymbol{K}_{\mathbf{1}}\mathbf{,}\boldsymbol{K}_{\mathbf{2}}\boldsymbol{\ll1}$

Examining the equation for $K_{12}^{PA}$, we find that in the limit of $K_{1}\ll1$:

$$\lim_{K_{1}\to0^{+}} K_{12}^{PA}=K_{1}\frac{K_{2}}{1+K_{2}}$$

However, if $K_{2}\ll1$ as well, then $K_{12}^{PA}\approx K_{1}K_{2}$, which implies that $C\to1$ in this region of parameter space (no cooperativity).

### Near-perfect adaptation for the FF only model

Given $K_{1},K_{2}$, for what value of $K_{12}$ do we hit the limit of NPA? In that case, we set $1/{z_{1}}=1/\left[ \left( 1+\varepsilon\right)z_{0} \right]$:

$$\frac{1+{x_{1}}/{K_{1}}+{y_{1}}/{K_{2}}+{x_{1}y_{1}}/{K_{12}}}{{x_{1}}/{K_{1}}}=\frac{1+{x_{0}}/{K_{1}}+{y_{0}}/{K_{2}}+{x_{0}y_{0}}/{K_{12}}}{\left( 1+\varepsilon\right){x_{0}}/{K_{1}}}$$

Again isolating the terms with $K_{12}$ onto the LHS:

$$\frac{K_{1}}{K_{12}}\left( y_{1}-\frac{y_{0}}{1+\varepsilon} \right)=\frac{1}{1+\varepsilon}\left( 1+\frac{K_{1}}{x_{0}}+\frac{K_{1}y_{0}}{K_{2}x_{0}} \right)-\left( 1+\frac{K_{1}}{x_{1}}+\frac{K_{1}y_{1}}{K_{2}x_{1}} \right)$$

OR:

$$K_{12}^{NPA}=\frac{K_{1}\left( y_{1}-\frac{y_{0}}{1+\varepsilon} \right)}{\frac{1}{1+\varepsilon}\left( 1+\frac{K_{1}}{x_{0}}+\frac{K_{1}y_{0}}{K_{2}x_{0}} \right)-\left( 1+\frac{K_{1}}{x_{1}}+\frac{K_{1}y_{1}}{K_{2}x_{1}} \right)}$$

OR:

$$K_{12}^{NPA}=K_{1}\frac{\left( y_{1}-\frac{y_{0}}{1+\varepsilon} \right)}{\frac{1}{1+\varepsilon}\left( \frac{1}{y_{0}}+\frac{K_{1}y_{0}}{K_{2}x_{0}} \right)-\left( \frac{1}{y_{1}}+\frac{K_{1}y_{1}}{K_{2}x_{1}} \right)}$$

OR:

$$K_{12}^{NPA}=K_{1}\frac{\left( y_{1}-\frac{y_{0}}{1+\varepsilon} \right)}{\frac{1/{y_{0}}}{1+\varepsilon}-\frac{1}{y_{1}}+\left( \frac{1}{1+\varepsilon}\frac{K_{1}y_{0}}{K_{2}x_{0}}-\frac{K_{1}y_{1}}{K_{2}x_{1}} \right)}$$

$C_{NPA+}$ and $C_{NPA-}$ can be derived by $C={K_{12}}/\left( K_{1}K_{2} \right)$ and setting $\varepsilon=+0.05$ or $\varepsilon=-0.05$, respectively.

### Regions I and II for the FF only model

Regions I and II can be defined as when the denominator of $K_{12}^{NPA}$ (for positive $\varepsilon$) becomes zero. Solving that relationship for $K_{2}$ gives a boundary (or boundaries) in the $K_{1}-K_{2}$ plane that demarcate Regions I and II:

$$K_{2}^{*}=\frac{\frac{1}{1+\varepsilon}\left( \frac{K_{1}y_{0}}{x_{0}} \right)-\left( \frac{K_{1}y_{1}}{x_{1}} \right)}{\left( 1+\frac{K_{1}}{x_{1}} \right)-\frac{1}{1+\varepsilon}\left( 1+\frac{K_{1}}{x_{0}} \right)}$$

Region I occurs when both the numerator and denominator of $K_{2}^{*}$ are positive. For Region I, there is an upper limit for feasible $K_{1}$, above which you cannot find a positive $K_{2}^{*}$, and that upper limit is where the denominator of $K_{2}^{*}$ is zero. Solving that relationship for $K_{1}$:

$$K_{1}^{upper}=\frac{\varepsilon}{\frac{1}{x_{0}}-\left( 1+\varepsilon\right)\frac{1}{x_{1}}}$$

Region II occurs when both the numerator and denominator of $K_{2}^{*}$ are negative. Therefore, there is a lower limit of $K_{1}$, below which you cannot find a positive $K_{2}^{*}$ for Region II, and that lower limit is where the numerator of $K_{2}^{*}$ is zero. Solving that relationship for $K_{1}$ requires unpacking $y_{0}$ and $y_{1}$.

$$K_{1}^{lower}=\frac{x_{1}-\left( 1+\varepsilon\right)x_{0}}{\varepsilon}$$

### Region I results from a simplification to the model for $\boldsymbol{K}_{\boldsymbol{1}}$ low, $\boldsymbol{K}_{\boldsymbol{2}}$ moderate

The model equations simplify when $K_{1}$ is small and $K_{2}$ is not. If $K_{1}$ is small, then $x/{K_{1}}$ swamps all denominators, resulting in a decoupled, relatively uninteresting set of equations:

$$\tau_{y}\frac{dy}{dt}=1-y$$

$$\tau_{z}\frac{dz}{dt}=1-z$$

These equations are (nearly) independent of $x$, and $y$ and $z$ are independent of each other. And the reason why it looks like NPA is because the equations are so insensitive to $x$. Simulations in this region of the parameter space do not experience a transient peak. Instead, the transient in $z$ is an exponential decay from the initial conditions to the final steady state. The vertical axis limits reveal there is a negligible percent change in the value of $z$ from $z_{0}$ to $z_{1}$ (the graph starts near 1 and ends up near 1), so that it technically fits the definition of NPA. See Fig. S1.

### Perfect adaptation in the FF/FB model

To derive the design rule for PA in the FF/FB model, we follow the same initial steps as for the FF only model, while recognizing that the constraint $z_{1}=z_{0}$ also implies $w_{1}=w_{0}=w$, as the value of $w$ is dependent only on $z$. Therefore, equating $1/{z_{1}}$ to $1/{z_{0}}$:

$$\frac{1+\frac{x_{1}}{K_{1}}+\frac{y_{1}}{K_{2}}+\frac{x_{1}y_{1}}{K_{12}}+\frac{w}{K_{3}}\left( 1+\frac{x_{1}}{K_{1}}+\frac{y_{1}}{K_{2}}+\frac{x_{1}y_{1}}{K_{12}} \right)}{\frac{x_{1}}{K_{1}}}=\frac{1+\frac{x_{0}}{K_{1}}+\frac{y_{0}}{K_{2}}+\frac{x_{0}y_{0}}{K_{12}}+\frac{w}{K_{3}}\left( 1+\frac{x_{0}}{K_{1}}+\frac{y_{0}}{K_{2}}+\frac{x_{0}y_{0}}{K_{12}} \right)}{\frac{x_{0}}{K_{1}}}$$

Isolating the terms with $K_{12}$ onto the LHS:

$$\frac{K_{1}}{K_{12}}\left( y_{1}+\frac{wy_{1}}{K_{3}}-y_{0}-\frac{wy_{0}}{K_{3}} \right)=\left( 1+\frac{K_{1}}{x_{0}}+\frac{K_{1}y_{0}}{K_{2}x_{0}}+\frac{K_{1}w}{K_{3}x_{0}}\left( 1+\frac{x_{0}}{K_{1}}+\frac{y_{0}}{K_{2}} \right) \right)-\left( 1+\frac{K_{1}}{x_{1}}+\frac{K_{1}y_{1}}{K_{2}x_{1}}+\frac{K_{1}w}{K_{3}x_{1}}\left( 1+\frac{x_{1}}{K_{1}}+\frac{y_{1}}{K_{2}} \right) \right)$$

Simplifying both sides:

$$\frac{K_{1}}{K_{12}}\left( 1+\frac{w}{K_{3}} \right)\left( y_{1}-y_{0} \right)=\left( \frac{K_{1}}{x_{0}}+\frac{K_{1}y_{0}}{K_{2}x_{0}}+\frac{w}{K_{3}}\left( \frac{K_{1}}{x_{0}}+\frac{K_{1}y_{0}}{K_{2}x_{0}} \right) \right)-\left( \frac{K_{1}}{x_{1}}+\frac{K_{1}y_{1}}{K_{2}x_{1}}+\frac{w}{K_{3}}\left( \frac{K_{1}}{x_{1}}+\frac{K_{1}y_{1}}{K_{2}x_{1}} \right) \right)$$

Further simplification on the RHS:

$$\frac{K_{1}}{K_{12}}\left( 1+\frac{w}{K_{3}} \right)\left( y_{1}-y_{0} \right)=\left( 1+\frac{w}{K_{3}} \right)\left( \frac{K_{1}}{x_{0}}+\frac{K_{1}y_{0}}{K_{2}x_{0}} \right)-\left( 1+\frac{w}{K_{3}} \right)\left( \frac{K_{1}}{x_{1}}+\frac{K_{1}y_{1}}{K_{2}x_{1}} \right)$$

Thus, the factors $\left( 1+w/{K_{3}} \right)$ drop out of the equation, which leaves us with Eq 4 from the text.

### Near-perfect adaptation in the FF/FB model

To derive the NPA constraint in the FF/FB model, we will once again set $1/{z_{1}}=1/\left[ \left( 1+\varepsilon\right)z_{0} \right]$, but this time in the case in which negative feedback is present:

$$\frac{1+{x_{1}}/{K_{1}}+{y_{1}}/{K_{2}}+{x_{1}y_{1}}/{K_{12}}+{w_{1}}/{K_{3}}\left( 1+{x_{1}}/{K_{1}}+{y_{1}}/{K_{2}}+{x_{1}y_{1}}/{K_{12}} \right)}{{x_{1}}/{K_{1}}}=\frac{1+{x_{0}}/{K_{1}}+{y_{0}}/{K_{2}}+{x_{0}y_{0}}/{K_{12}}+{w_{0}}/{K_{3}}\left( 1+{x_{0}}/{K_{1}}+{y_{0}}/{K_{2}}+{x_{0}y_{0}}/{K_{12}} \right)}{\left( 1+\varepsilon\right){x_{0}}/{K_{1}}}$$

Again isolating the terms with $K_{12}$ onto the LHS:

$$\frac{K_{1}}{K_{12}}\left( y_{1}\left( 1+\frac{w_{1}}{K_{3}} \right)-\frac{y_{0}}{\left( 1+\varepsilon\right)}\left( 1+\frac{w_{0}}{K_{3}} \right) \right)=\frac{1}{\left( 1+\varepsilon\right)}\left( 1+\frac{K_{1}}{x_{0}}+\frac{K_{1}y_{0}}{K_{2}x_{0}}+\frac{w_{0}}{K_{3}}\left( 1+\frac{K_{1}}{x_{0}}+\frac{K_{1}y_{0}}{K_{2}x_{0}} \right) \right)-\left( 1+\frac{K_{1}}{x_{1}}+\frac{K_{1}y_{1}}{K_{2}x_{1}}+\frac{w_{1}}{K_{3}}\left( 1+\frac{K_{1}}{x_{1}}+\frac{K_{1}y_{1}}{K_{2}x_{1}} \right) \right)$$

OR:

$$K_{12}^{NPA,FB}=\frac{K_{1}\left( y_{1}-\frac{Ay_{0}}{\left( 1+\varepsilon\right)} \right)}{\frac{A}{\left( 1+\varepsilon\right)}\left( 1+\frac{K_{1}}{x_{0}}+\frac{K_{1}y_{0}}{K_{2}x_{0}} \right)-\left( 1+\frac{K_{1}}{x_{1}}+\frac{K_{1}y_{1}}{K_{2}x_{1}} \right)}$$

where $A=\left( 1+\frac{w_{0}}{K3} \right)/\left( 1+\frac{w_{1}}{K3} \right)$. This formula leaves us with the question of the values of $w_{1}$ and $w_{0}$, which depend on the value of $K_{12}$.

### Regions I and II in the FF/FB model

As before, Regions I and II can be found by setting the denominator of $K_{12}^{NPA,FB}$ to zero. However, we additionally must evaluate $w_{0},w_{1}$ at $K_{12}\to\infty$. Given the denominator is equivalent to the one for FFL only, with the transformation of $1/\left( 1+\varepsilon\right)\to A/\left( 1+\varepsilon\right)$, we arrive at

$$K_{2}^{*,FB}=\frac{\frac{A}{1+\varepsilon}\left( \frac{K_{1}y_{0}}{x_{0}} \right)-\left( \frac{K_{1}y_{1}}{x_{1}} \right)}{\left( 1+\frac{K_{1}}{x_{1}} \right)-\frac{A}{1+\varepsilon}\left( 1+\frac{K_{1}}{x_{0}} \right)}$$

Note that in this formulation, $A$ also depends on the value of $K_{2}$, so this is not an explicit solution. As mentioned in the main body text (see Fig. 3E), Region I in the FF/FB case is expanded compared to the FF only case. However, even though the boundary for Region II (FF/FB) nearly overlaps that of the FF case, they are not identical.

### Model equations with cooperativity between X and W, and/or between Y and W

To include additional cooperativity factors, the model differential equations retain the same structure (Eqs. 2-3,6-7). The changes are in the activation function for Z, which includes factors $C_{13}$ and $C_{23}$, which control the cooperativity between X and W, and between Y and W, respectively.

$$f_{z}\left( \frac{x}{K_{1}},\frac{y}{K_{2}},\frac{w}{K_{3}} \right)=\frac{\frac{x}{K_{1}}}{1+\frac{x}{K_{1}}+\frac{y}{K_{2}}+\frac{xy}{\left( CK_{1}K_{2} \right)}+\frac{w}{K_{3}}+\frac{xw}{\left( C_{13}K_{1}K_{3} \right)}+\frac{yw}{\left( C_{23}K_{2}K_{3} \right)}+\frac{xyw}{\left( CC_{13}C_{23}K_{1}K_{2}K_{3} \right)}}$$

### Model equations and perfect adaptation for $\boldsymbol{n=2}$

If the Hill coefficient is set to $n=2$, the model equations remain the same, except the activation functions change. For example, the activation function for $y$ becomes:

$$f_{y}\left( \frac{x}{K_{1}} \right)=\frac{\left( x/{K_{1}} \right)^{n}}{1+\left( x/{K_{1}} \right)^{n}}$$

The changes to the other activation functions are analogous.

To derive PA for the $n=2$ model, the first steps of the analysis for $n=1$ apply, but upon equating $1/{z_{1}}$ to $1/{z_{0}}$, we have:

$$\frac{1+\left( {x_{1}}/{K_{1}} \right)^{n}+\left( {y_{1}}/{K_{2}} \right)^{n}+\left( {x_{1}y_{1}}/{K_{12}} \right)^{n}}{\left( {x_{1}}/{K_{1}} \right)^{n}}=\frac{1+\left( {x_{0}}/{K_{1}} \right)^{n}+\left( {y_{0}}/{K_{2}} \right)^{n}+\left( {x_{0}y_{0}}/{K_{12}} \right)^{n}}{\left( {x_{0}}/{K_{1}} \right)^{n}}$$

Isolating the terms with $K_{12}$ onto the LHS:

$$\left( \frac{K_{1}}{K_{12}} \right)^{n}\left( y_{1}^{n}-y_{0}^{n} \right)=\left( 1+\left( \frac{K_{1}}{x_{0}} \right)^{n}+\left( \frac{K_{1}y_{0}}{K_{2}x_{0}} \right)^{n} \right)-\left( 1+\left( \frac{K_{1}}{x_{1}} \right)^{n}+\left( \frac{K_{1}y_{1}}{K_{2}x_{1}} \right)^{n} \right)$$

OR:

$$\left( K_{12}^{PA} \right)^{n}=\frac{y_{1}^{n}-y_{0}^{n}}{\frac{1}{x_{0}^{n}}\left( 1+\left( \frac{y_{0}}{K_{2}} \right)^{n} \right)-\frac{1}{x_{1}^{n}}\left( 1+\left( \frac{y_{1}}{K_{2}} \right)^{n} \right)}$$

### Derivation of the principal eigenvalue

Linearizing the model equations about the steady state results in:

$$\tau_{y}\frac{d\hat{y}}{d\tau}=J_{1}\hat{F}-\hat{y}$$

$$\tau_{z}\frac{dz}{dt}=J_{2}\hat{F}+J_{3}\hat{y}+J_{4}\hat{w}-\hat{z}$$

$$\tau_{w}\frac{dw}{dt}=J_{5}\hat{z}-\hat{w}$$

…where the $J_{i}$’s are Jacobian matrix elements. The “hatted” variables are deviation variables; for example, $\hat{z}=z-z_{0}$, where $z_{0}$ is the steady state about which we are expanding. The deviation format of the other variables are analogous.

Laplace transforming this set of equations:

$$Y\left( s \right)=\frac{J_{1}}{\tau_{y}s+1}F\left( s \right)e^{-\theta_{y}s}$$

$$\left( \tau_{z}s+1 \right)Z\left( s \right)=\left[ J_{2}F\left( s \right)+J_{3}Y\left( s \right)+J_{4}W\left( s \right) \right]e^{-\theta_{z}s}$$

$$W\left( s \right)=\frac{J_{5}}{\tau_{w}s+1}Z\left( s \right)e^{-\theta_{w}s}$$

Substituting $Y\left( s \right)$ and $W(s)$ into this expression for $Z\left( s \right)$:

$$\left( \tau_{z}s+1 \right)Z\left( s \right)=\left[ J_{2}F\left( s \right)+\frac{J_{1}J_{3}}{\tau_{y}s+1}F\left( s \right)e^{-\theta_{y}s}+J_{4}\frac{J_{5}}{\tau_{w}s+1}Z\left( s \right)e^{-\theta_{w}s} \right]e^{-\theta_{z}s}$$

OR:

$$\left( \tau_{z}s+1-\frac{J_{4}J_{5}}{\tau_{w}s+1}e^{-(\theta_{w}+\theta_{z})s} \right)Z\left( s \right)=\left[ J_{2}F\left( s \right)+\frac{J_{1}J_{3}}{\tau_{y}s+1}F\left( s \right)e^{-\theta_{y}s} \right]e^{-\theta_{z}s}$$

In other words:

$$G\left( s \right)\equiv\frac{Z\left( s \right)}{F\left( s \right)}=\frac{J_{2}+\frac{J_{1}J_{3}}{\tau_{y}s+1}e^{-\theta_{y}s}}{\left( \tau_{z}s+1-\frac{J_{4}J_{5}}{\tau_{w}s+1}e^{-(\theta_{w}+\theta_{z})s} \right)e^{\theta_{z}s}}$$

Simplifying the denominators:

$$G\left( s \right)=\frac{\left( \tau_{w}s+1 \right)\left( J_{2}\left( \tau_{y}s+1 \right)+J_{1}J_{3}e^{-\theta_{y}s} \right)}{\left( \tau_{y}s+1 \right)\left( \left( \tau_{w}s+1 \right)\left( \tau_{z}s+1 \right)-J_{4}J_{5}e^{-(\theta_{w}+\theta_{z})s} \right)e^{\theta_{z}s}}$$

Therefore, the characteristic equation is:

$$C\left( s \right)=\left( \tau_{y}s+1 \right)\left( \left( \tau_{w}s+1 \right)\left( \tau_{z}s+1 \right)-Je^{-(\theta_{w}+\theta_{z})s} \right)e^{\theta_{z}s}=0$$

…where $J=J_{4}J_{5}$. Finding the complex roots, $s$ that make $C\left( s \right)=0$ will give us our eigenvalues. This analysis means that only two partial derivatives are needed ($J_{4}$ and $J_{5}$).

In the case in which there is no FF loop, the characteristic equation becomes:

$$C\left( s \right)=\left( \left( \tau_{w}s+1 \right)\left( \tau_{z}s+1 \right)-Je^{-(\theta_{w}+\theta_{z})s} \right)e^{\theta_{z}s}=0$$

In other words, the only difference in the structure of the characteristic equation is the $\left( \tau_{y}s+1 \right)$ factor. However, it should be noted that the values of $J_{4}$ and $J_{5}$ will also typically be different between FB only and FF/FB.

The zeros of $e^{\theta_{z}s}$ are always eigenvalues, but they are uninteresting for our purposes. For the FF/FB system, $-1/{\tau_{y}}$ is also an eigenvalue. To solve for the principal eigenvalue, we remove these factors, which leaves us with the expression inside the parentheses:

$$\left( \tau_{w}s+1 \right)\left( \tau_{z}s+1 \right)-Je^{-\left( \theta_{w}+\theta_{z} \right)s}=\tau_{w}\tau_{z}s^{2}+\left( \tau_{w}+\tau_{z} \right)s+1-Je^{-\left( \theta_{w}+\theta_{z} \right)s}$$

To solve this, we will split into the real and imaginary parts. First, substituting $s=\alpha+i\omega$.

$$\tau_{w}\tau_{z}\left( \alpha+i\omega\right)^{2}+\left( \tau_{w}+\tau_{z} \right)\left( \alpha+i\omega\right)+1-Je^{-\left( \theta_{w}+\theta_{z} \right)\alpha}\left( \cos\left( \left( \theta_{w}+\theta_{z} \right)\omega\right)-i\sin\left( \left( \theta_{w}+\theta_{z} \right)\omega\right) \right)$$

Therefore, equation for the real part is:

$$\tau_{w}\tau_{z}\left( \alpha^{2}-\omega^{2} \right)+\left( \tau_{w}+\tau_{z} \right)\alpha+1-Je^{-\left( \theta_{w}+\theta_{z} \right)\alpha}\cos\left( \left( \theta_{w}+\theta_{z} \right)\omega\right)=0$$

And the equation for the imaginary part is:

$$2\tau_{w}\tau_{z}\alpha\omega+\left( \tau_{w}+\tau_{z} \right)\omega+Je^{-\left( \theta_{w}+\theta_{z} \right)\alpha}\sin\left( \left( \theta_{w}+\theta_{z} \right)\omega\right)=0$$

Therefore, the principal eigenvalue is dependent only on the time scale parameters of the model, $\tau_{w},\tau_{z},\theta_{w},\theta_{z}$, and the value of $J$. As we have set all $\tau_{i}$’s equal to one, and all $\theta_{i}$’s equal to one half, we can solve these equations to find the values of $\alpha,\omega$ as functions of all feasible values of $J$. Afterwards, computation of the real part of the principal eigenvalue for any parameter set $\left\{ K_{i} \right\}$ requires only the computation of $J$ for that parameter set and mapping this onto $\alpha\left( J \right)$.

### Analysis of RegulonDB data set

To analyze the RegulonDB data set, we first created two look-up tables: one between the names of TF complexes and the genes that encode their constituent proteins, and one between the names of genes that code for proteins that participate in at least one TF complex and the TF complexes those proteins participate in. Next, we created a matrix, $M$, with each row corresponding to a TF complex (a total of 207 rows), and each column to a target of the TF complex. The targets were split into two categories. The first category consisted of the TF complexes themselves, and they occupied the first 207 columns of the matrix. The remaining columns represented genes that were targets of the list of TF complexes, but did not code for proteins that participated in any of the 207 TF complexes. Therefore, $M$ consisted of two submatrices: the square, 207-by-207 matrix $M_{TF}$, which contained the cross-regulatory information regarding the TF networks in E. coli; and the rectangular matrix, $M_{targets}$, which contained the information of the end-nodes of the gene regulatory networks.

After creating $M_{TF}$, we searched for pairs of off-diagonal elements that participate in feedback loops of length 2, as explained in the main-text Methods section. We found 17 such cases, of which 5 were ruled out as positive feedback loops. The remaining 12 were individually tested to determine if the node corresponding to the activator of the negative feedback loop (Z) was also the endpoint of an I1-FFL. To do this, six further criteria must be satisfied:

1. Z must be regulated positively by one element (X) and negatively by another (Y).
2. Y must be positively regulated by X
3. X must not be regulated by Y, Z, or W (the element of the negative feedback loop activated by Z).
4. Neither Z nor W could regulate Y.
5. Neither X nor Y could regulate W.
6. Autoregulation is allowed.

These six criteria can be summed up in the following submatrix of $M_{TF}$, where the rows and columns correspond to X,Y,Z,W, in that order:

$$\left[ \begin{matrix} - & 1 & 1 & 0 \\ 0 & - & -1 & 0 \\ 0 & 0 & - & 1 \\ 0 & 0 & -1 & - \end{matrix} \right]$$

…where a “2” could also stand in place of either a “+1” or a “-1”, the zeros must be zeros, and the diagonal elements (autoregulation) could be any value. If these were satisfied, then the motif consisting of X, Y, Z, and W was classified as a FF/FB structure. Two such structures were found in the *E. coli* RegulonDB data set, both consisting of X = Ada, Z = FlhDC, and W = FliZ. Further information can be found in Table S1.

# Supplementary Figures


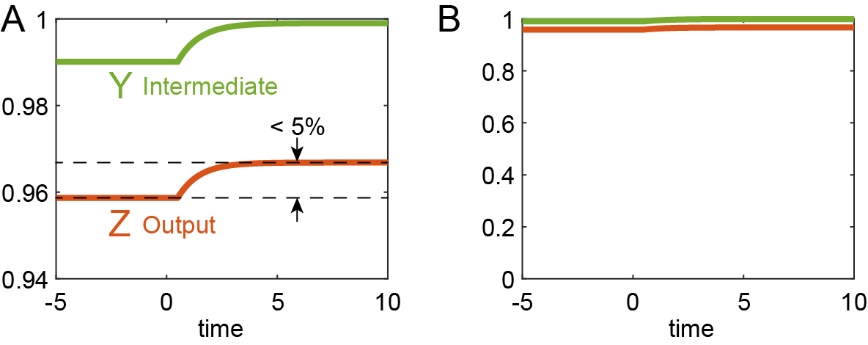


**Fig. S1: Dynamic behavior of the model in Region I**

1. In Region I, Z is relatively insensitive to both X and Y. Therefore, the change in Z that is sparked by a change in X is always less than 5%, regardless of the value of the cooperativity value.
2. Plot from (A) with a vertical axis scale from zero to one, to illustrate how little the model changes with respect to a change in X.


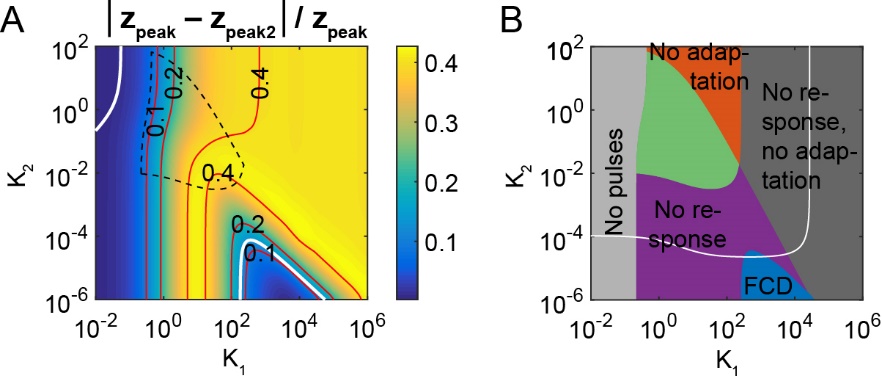


**Fig. S2: Analysis of the phenotypes in the** $\boldsymbol{K}_{\boldsymbol{1}}\boldsymbol{,}\boldsymbol{K}_{\boldsymbol{2}}$ **plane.**

1. Measure of the ability of the model to act as a fold-change detector. The metric, $\left| z_{peak}-z_{peak,2} \right|/{z_{peak}}$, measures the relative difference between two peaks in $z$: one in which the input concentration changes from 1 to 10 ($z_{peak}$), and the other in which it changes from 5 to 50 ($z_{peak,2}$). Both have a fold-change of 10. The white curves correspond to Regions I and II from Fig. 2. Note that Region II is where FCD was found in Goentoro et al., 2009. Indeed, the criterion used by Goentoro et al. is that this metric should be less than 0.1 for the I1-FFL to act as an FCD, which almost perfectly coincides with Region II. The region enclosed by the black dashed curve is the same biologically realistic region as found in Fig. 2. Using this region as a reference, it can be seen that FCD is also possible when the relative pulse amplitude is diminished (i.e., $P<0.1$).
2. Different phenotypes found in the $K_{1},K_{2}$ plane. For low values of $K_{1}$, pulses are not found (light gray region; $P<0.1$). In the rust-colored region, the cooperativity required for PA is overly-high ($C_{PA}<0.01$). In the purple-colored region, there is too little absolute response ($z_{peak}<0.01$). For high values of $K_{1}$ (dark gray region), there is neither adaptation, or an appreciable absolute response. In the blue region, the I1-FFL can act as an FCD (see part A); however, this region resides in the portion of the plane in which $z_{peak}<0.01$. In fact, $z_{peak}<{10}^{-4}$ (white curve) for almost the entirety of the FCD region.


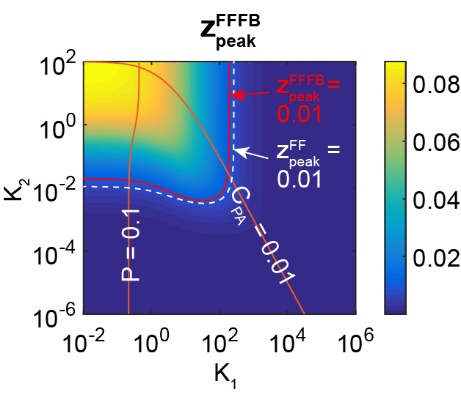


**Fig. S3: Heatmap of** $\boldsymbol{z}_{\boldsymbol{peak}}$ **for the FF/FB model**

This heatmap (and the corresponding contour for $z_{peak}^{FFFB}=0.01$, red curve) shows the biologically realistic region for the FF/FB model is nearly identical to that for the FF only model. The contour for the FF only model ($z_{peak}^{FF}=0.01$) is shown as a dashed white curve. We have also plotted the contours for P = 0.1 and $C_{PA}=0.01$, which are the same contours as in the FF only model. Together, these three contours demarcate the biologically realistic region.


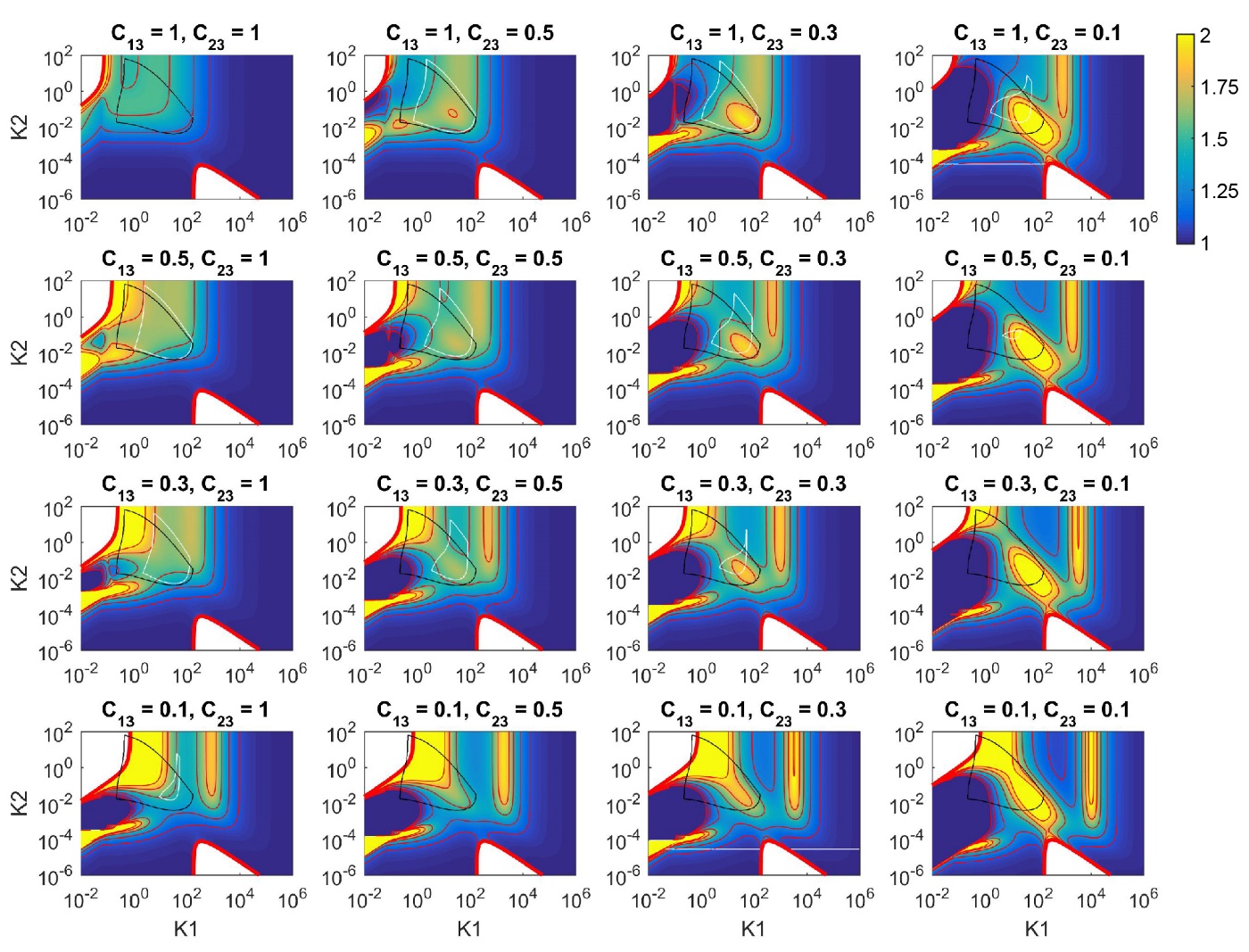


**Fig. S4: Adding cooperativity to interactions between X and W, and/or between Y and W.** Each of the heatmaps depicted here is the same as that found in Fig. 3F (i.e., ${\Delta C_{FFFB}}/{\Delta C_{FF}}$), but for different values of $C_{13}$ and $C_{23}$. The titles above each heatmap denote the values of $C_{13}$ and $C_{23}$. Varying these resulted in two changes to the heatmap. First, as cooperativity increases (for either parameter), the “biologically realistic” region (enclosed by white curve) decreases in area (compare to area enclosed by black curve, which is the biologically realistic region for no cooperativity). At the most extreme, this region disappears. Second, the improvement in robustness of the NPA phenotype increases, as can be seen by the yellow colors appearing in the biologically realistic region. Therefore, some measure of advantage may come with cooperativity, but this may be offset by the fragility of the shrinking biologically realistic region. Note that the upper-left heatmap is the same as the one found in Fig. 3F.


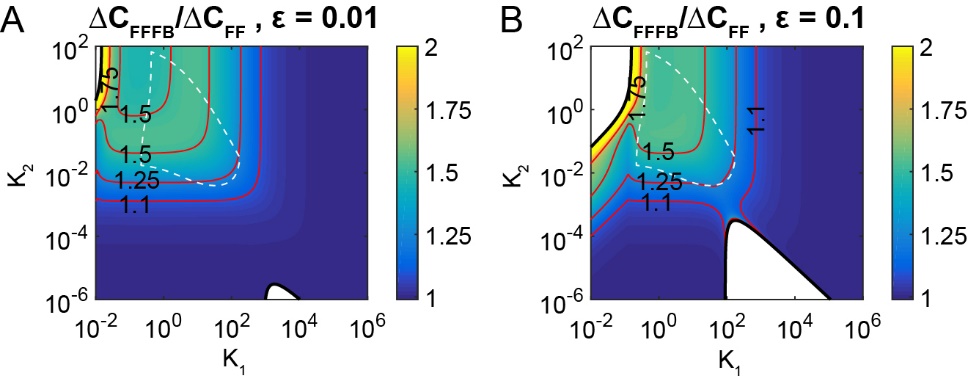


**Fig. S5: Varying** $\boldsymbol{\varepsilon}$

1. Same heatmap as in Fig. 3F, but for $\varepsilon=0.01$.
2. Same heatmap as in Fig. 3F, but for $\varepsilon=0.1$.


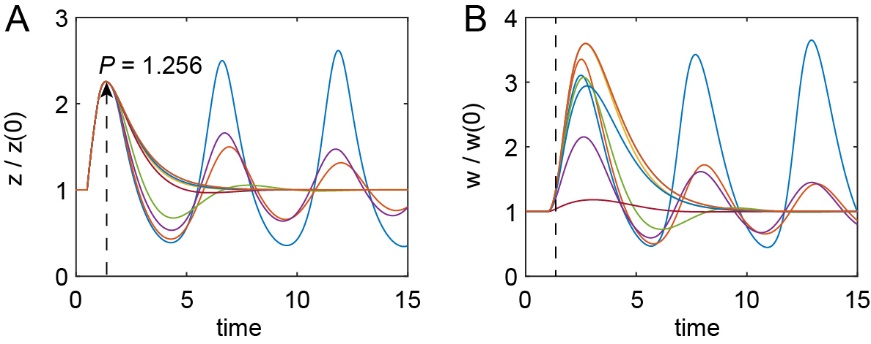


**Fig. S6: Independence of** $\boldsymbol{P}$ **from** $\boldsymbol{K}_{\boldsymbol{3}}\boldsymbol{,}\boldsymbol{K}_{\boldsymbol{4}}$ **for the FF/FB model**

1. Plot of normalized curves for the output, $z$, for various values of $K_{3},K_{4}$ and for $K_{1}=1,K_{2}=0.1,C=C_{PA}$. Note that each curve, regardless of the values of $K_{3},K_{4}$, has the same initial transient and same peak value ($P=1.256$) and peak time, even the unstable systems.
2. Plot of normalized curves for the negative feedback node, $w$, for the same parameter sets as in (A). Vertical dashed line denotes the peak time. Note that, at the time when the peak is achieved, the value of $w$ has changed only negligibly, which explains why the value of $P$ in the FF/FB model is insensitive to values of the negative feedback parameters, $K_{3},K_{4}$. This also implies the peak is strictly due to the action of the FF loop.
